# Supplementary material for: Functional and Structural Properties of Interhemispheric Interaction between Bilateral Precentral Hand Motor Regions in a Top Wheelchair Racing Paralympian
Source: Brain Sci. 2023 Apr 25;13(5):715. doi: 10.3390/brainsci13050715 (PMC10216139; doi:10.3390/brainsci13050715)
Supplement: Supplementary file 1 [file brainsci-13-00715-s001.zip › brainsci-2314545-supplementary.pdf]

Supplementary Methods and Results

fMRI experiment on a right-finger task

Using the same instrument (MAGNETOM Trio Tim with a 32-channel array coil at NICT CiNet; Siemens, Germany) as in the main fMRI experiment, we obtained fMRI data, while all participants except four control participants performed a right-finger task in another experimental run. Each participant completed one experimental run for this task. The time schedule for the run (15s ON-Off design, 160 s in total), auditory stimuli (1 Hz tones), and instructions given during the run were identical to those in the right-hand task. During the task epochs, the participants pressed an MR-compatible button (Current Design Inc., Philadelphia, PA, USA) using their right index finger precisely in synchrony with 1-Hz cyclic tones (Supplementary Figure 3A). They were asked to press a button repetitively without releasing the finger from the button. In addition, we asked the participants to close their eyes just before the start of the experiment and to keep their eyes closed throughout the run. The MRI scan parameters and image preprocessing were identical to those used in the right-hand task.

After preprocessing, in the single-subject analysis, we evaluated the task-related activity using a general linear model for each participant. The design matrix contains a boxcar function for the task epoch, which is convolved using a canonical hemodynamic response function. To correct the residual motion-related variance after realignment, we included six realignment parameters in the design matrix as regressors of no interest. In the analysis, global mean scaling was not performed to avoid inducing Type I errors in the evaluation of negative BOLD responses. We generated an image showing the task-related activity for each participant, which was used in the subsequent analyses. Furthermore, we identified the significant activation and deactivation of the ROIs (see Materials and Methods section 2.6.2) for each paraplegic participant (Supplementary Figure 3B). In the second-level analyses, we performed a one-sample t-test to identify the activation and deactivation of ROIs in the control group (Holmes & Friston, 1998).

We also directly compared the task-related activity obtained from each paraplegic participant to that of the control participants to determine whether there were significant clusters of voxels within the left and right ROIs in which a paraplegic participant had stronger activity than the control participants. To assess statistical differences, we used Crawford and Howell's t-test (see Materials and Methods in the main text). In this analysis, we included the age and sex of all participants as nuisance covariates (effect of no interest) because these factors could influence the evaluation of the present between-group differences. When a significant cluster in the right ROI was found, we extracted the parameter estimate of activity from the cluster for each participant, plotted it, and calculated Cohen's d to assess the effect size of the difference.

The results are shown in Supplementary Figure 3. P1 showed a significant cluster of active voxels in the right (ipsilateral) ROI (Supplementary Figure 3B), which was not observed in the other paraplegic participants (P2-P4) and the control group. All paraplegic participants showed a significant cluster of active voxels in the left (contralateral) ROI, similar to the control group (Supplementary Figure 3B). Thus, only P1 showed activation in the bilateral precentral hand sections during the right-finger task, as observed during the right-hand task (Figure 1).

When we directly compared the brain activity of each paraplegic participant with that of the control participants, only P1 showed a significant cluster of voxels (22 voxels; $p < 0.001$ corrected) with greater activity in the right ROI than in the control group (yellow section in Supplementary Figure 3C). This cluster was in the anterior part of the right ROI, which partly overlapped with the region in which P1 showed greater activity than control participants during the right-hand task (Figure 3A). When we examined the data obtained from the identified cluster, P1 showed greater activity than control participants, and the value deviated by more than five SDs from the control mean (Supplementary Figure 3D; $d = 5.36$). In contrast, the data obtained from the other paraplegic participants were within the range of those obtained from the control participants.

Supplementary Figures

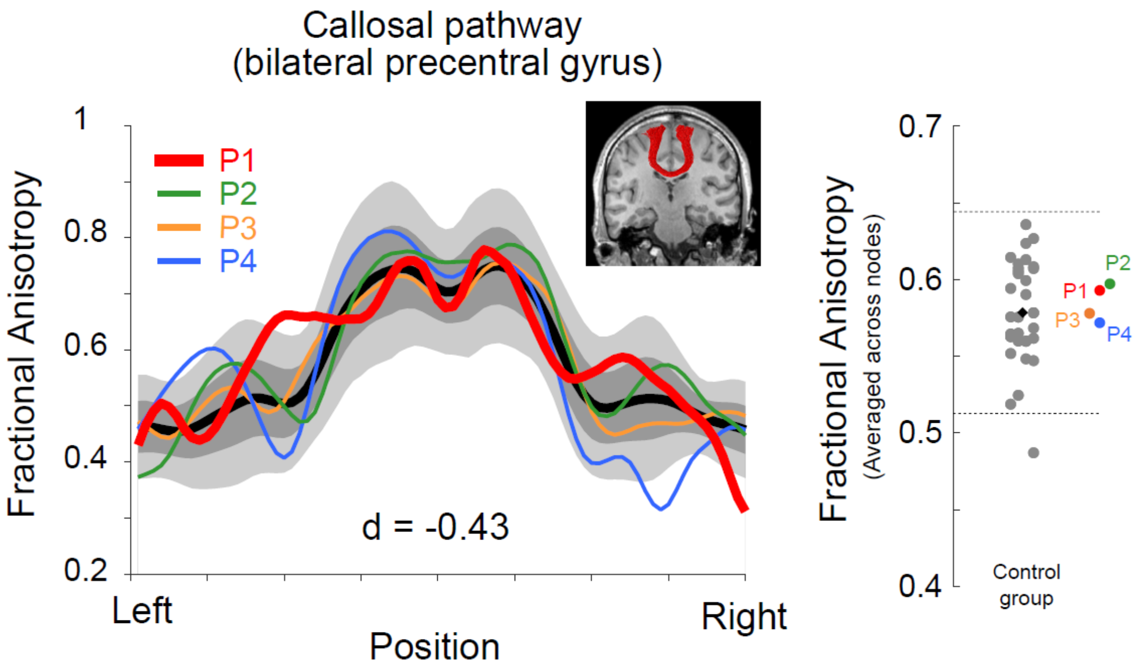

Supplementary Figure 1. Tract profile of fractional anisotropy (FA) along the transcallosal pathway connecting the bilateral precentral gyrus (red U-shaped curved lines in the upper figure), and the FA in each participant averaged across nodes. The conventions are identical to those in Figure 4.

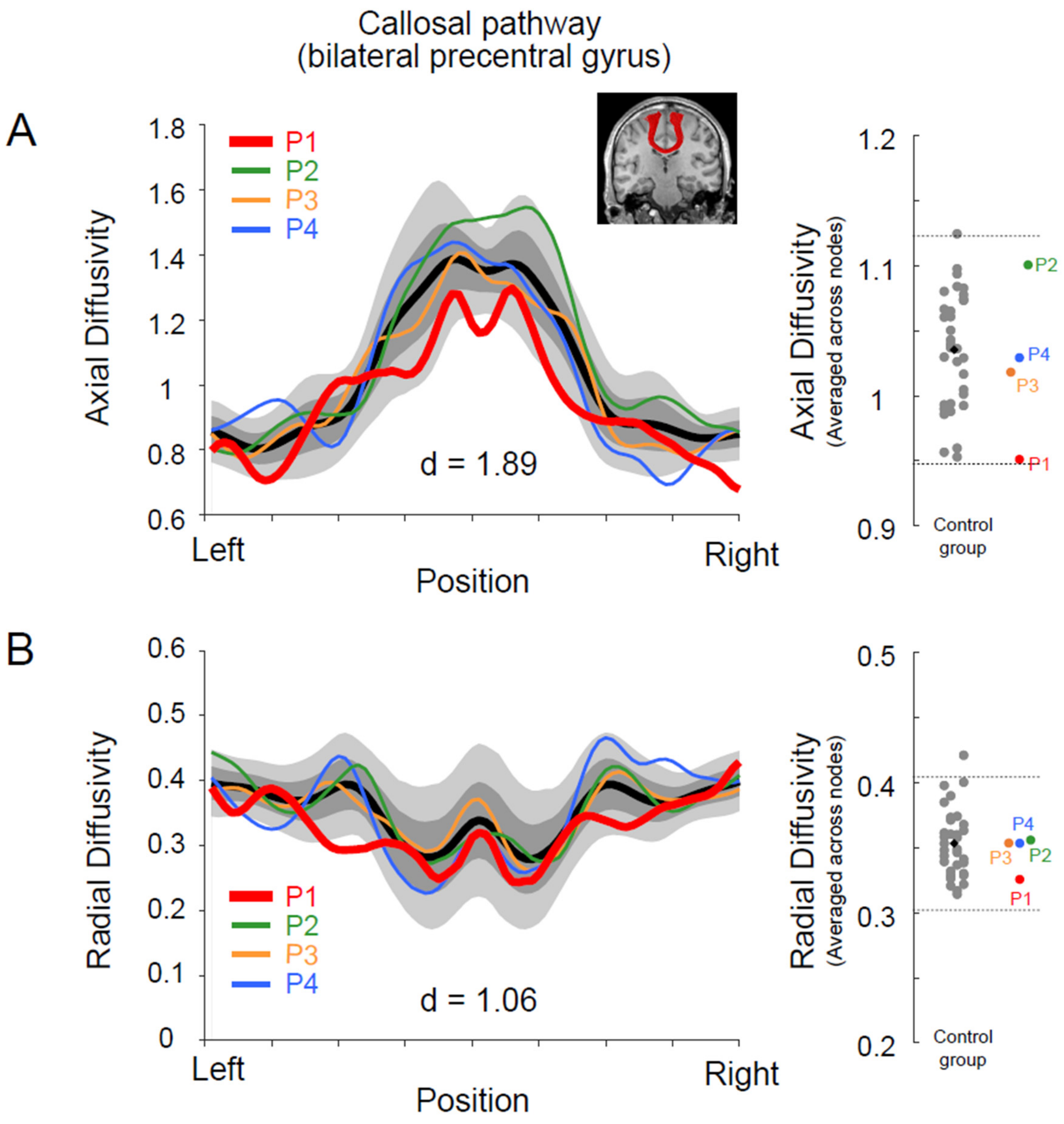

Supplementary Figure 2. (A) Tract profile of axial diffusivity (AD; unit of diffusivity, μm^2/ms) along the transcallosal pathway connecting the bilateral precentral gyrus (red U-shaped curved lines in the upper figure), and the AD in each participant averaged across nodes. (B) Tract profile of radial diffusivity (RD; unit of diffusivity, μm^2/ms) along the pathway, and the RD in each participant averaged across nodes. The conventions are identical to those in Figure 4.

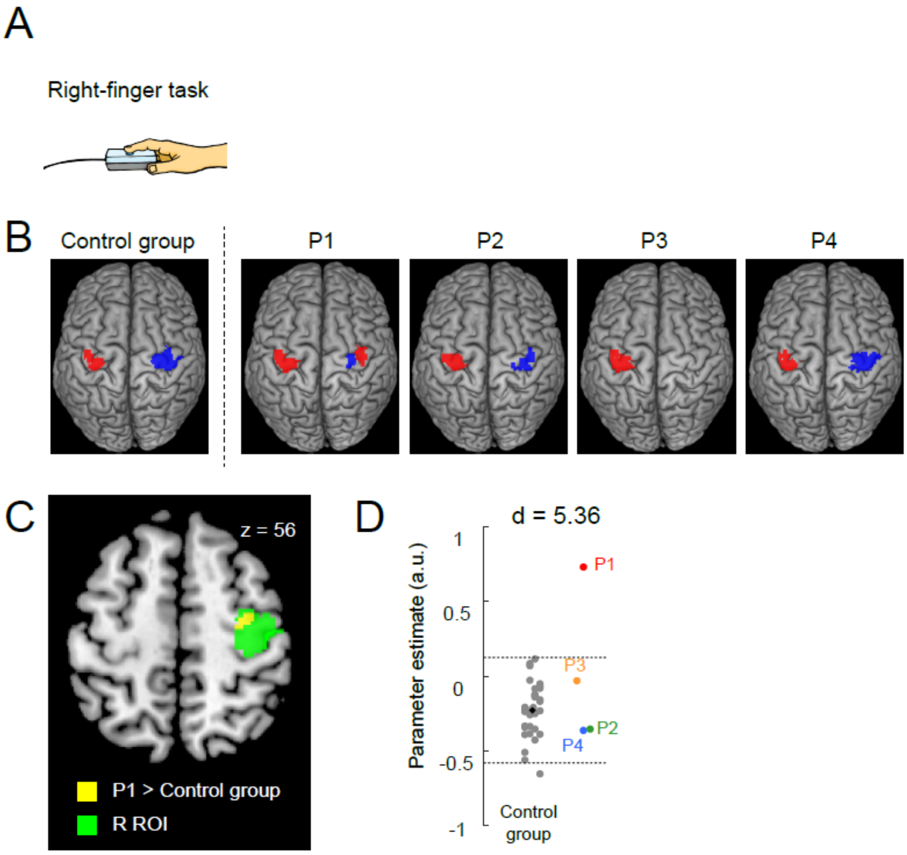

Supplementary Figure 3. Task and results of data from the right-finger task. A: Schematic illustration of the task. B: Bilateral activation (red) and deactivation (blue) in the left and right ROIs obtained from the control group (leftmost panel) and each paraplegic participant (P1-P4) during the right-finger task. C: P1 demonstrates a significant cluster of voxels showing greater activity than the control group (yellow sections). Green section indicates the right ROI. D: Individual parameter estimates of the activity obtained from the identified cluster in the right ROI (yellow section in panel C). The conventions are identical to those in Figure 2B. Abbreviations: ROI, region-of-interest; R, right; MNI, Montreal Neurological Institute; SD, standard deviation.
